# Comparison between drug-coated balloons and drug-eluting stents in very small coronary artery interventions

**DOI:** 10.1038/s41598-022-14047-7

**Published:** 2022-06-23

**Authors:** Cheng-Hsuan Tsai, Chih-Fan Yeh, Shih-Wei Meng, Chi-Sheng Hung, Mao-Shin Lin, Ching-Chang Huang, Chun-Kai Chen, Kuo-Ping Huang, Ying-Hsien Chen, Hsien-Li Kao

**Affiliations:** 1 National Taiwan University College of Medicine, Graduate Institute of Clinical Medicine, Taipei, Taiwan; 2 Department of Internal Medicine, National Taiwan University Hospital Jinshan Branch, New Taipei City, Taiwan; 3 Division of Cardiology, Department of Internal Medicine and Cardiovascular Center, National Taiwan University Hospital, 7 Chung-Shan South Road, 100 Taipei, Taiwan; 4 Cardiovascular Center, National Taiwan University Hospital, Taipei, Taiwan; 5 Division of Cardiology, Department of Internal Medicine, National Taiwan University Hospital, Hsin-Chu Branch, Hsin-Chu, Taiwan

**Keywords:** Interventional cardiology, Cardiac device therapy

## Abstract

The optimal management of very small vessel (reference diameter from 2.0 to 2.25 mm) in percutaneous coronary interventions (PCIs) is controversial. We aimed to compare the efficacy and safety of drug-coated balloons (DCBs) and drug-eluting stents (DESs) for de-novo very small vessel interventions. We conducted a retrospective analysis of consecutive patients who received very small vessel PCI with a DCB or DES between January 2018 and March 2021. The outcome measures were the incidence of ischemia-driven target lesion revascularization (TLR) and major adverse cardiac and cerebrovascular events (MACCEs) within 1 year after PCI. MACCEs were defined as the composite of ischemia-driven TLR, all-cause death, non-fatal acute coronary syndrome, stroke, or heart failure requiring hospitalization. A total of 205 patients undergoing PCI with a DCB or DES were enrolled in this study. The procedural complication rate was 2.5% in the DES group and 1.7% in the DCB group (P = 1.000). After 1-year of follow-up, the cumulative incidence of TLR was 7.2% in the DCB group and 4.9% in the DES group (P = 0.530). The cumulative incidence of MACCEs was 10.6% in the DCB group and 12.7% in the DES group (P = 0.769). Only female gender, acute coronary syndrome on presentation, and dual antiplatelet therapy duration < 3 months were significantly associated with MACCEs at 1 year, but the use of DCB or DES was not. The use of DCBs or DESs in de novo very small vessel intervention was not associated with different outcomes at 1 year.

## Introduction

Percutaneous coronary intervention (PCI) in small vessels (reference diameter < 2.5 mm) for coronary artery disease is a challenge for interventional cardiologists due to a low success rate and high rates of restenosis and adverse cardiovascular events during follow up^1–3^. Currently, there are no consensus or guidelines for interventions in small vessels, and the decision is usually left to the operator. The optimal choice of therapeutic modality for very small (reference vessel diameter, RVD: 2.0–2.25 mm) vessels is even less certain^4,5^.

Although ultrathin-strut drug-eluting stents (DESs) have been shown to outperform previous generations of DESs in small vessel PCI, their use in very small vessel is unproven^6^. Drug-coated balloons (DCBs) are initially used to treat in-stent restenosis^7^, however they have also been applied in de novo small vessel PCI^8,9^. DCBs may prevent neo-intimal formation without leaving permanent implant thickness in the vessel segment^9^, and thus they may be ideal for very small vessels. A recent study demonstrated that DCBs were noninferior to DESs with regards to 9-month in-segment restenosis for vessels ranging from 2.25–2.75 mm in diameter^5^, however very few studies have directly compared DCBs and DESs in very small vessel disease. Therefore, in this study, we performed a retrospective analysis on the safety and efficacy of DCBs versus DESs in very small vessel PCI.

## Methods

### Patients and target vessels

In this retrospective cohort study, we reviewed all patients receiving PCI from January 2018 to March 2021 at National Taiwan University Hospital. The inclusion criteria included (1) patients received PCI involving at least one de novo very small vessel lesion, defined as an RVD 2.0–2.25 mm, treated with either a DCB or DES; (2) the very small vessel was measured using quantitative coronary angiography (QCA), intravascular ultrasound (IVUS), or optical coherence tomography (OCT) during the index procedure. The decision on whether to use a DCB or DES was made by the operator and the adjunctive pre-treatment with semi-compliant, noncompliant, or scoring/cutting balloon angioplasties were allowed.

Clinical information was reviewed through electronic medical records from the National Taiwan University Hospital database, including baseline demographics, past medical history, complete blood counts, biochemistry studies, and medications after the index procedure. Coronary angiograms, procedural details, procedural results and complications were carefully collected and reviewed by independent interventionists. This retrospective study was approved by the Institutional Review Board of National Taiwan University Hospital, and this study was performed in accordance with relevant guidelines and regulations. The need of informed consent has been waived by the Institutional Review Board of National Taiwan University Hospital (202008020RINC).

### Study devices

The available DCBs at our institution with nominal diameters of 2.0 mm and 2.25 mm for very small vessel lesions were SeQuent Please, SeQuent Please Neo (B. Braun, Berlin, Germany), and Agent (Boston Scientific, Wurselen, Germany). The available DESs with stent diameters of 2.0 mm and 2.25 mm for very small vessel lesions were sirolimus-eluting Orsiro (Biotronik, Buloch, Switzerland), everolimus-eluting Synergy (Boston Scientific, Galway, Ireland), Xience Alpine (Abbott Vascular, Tipperary, Ireland), and zotarolimus-eluting Resolute Onyx (Medtronic, Galway, Ireland). The choice of device was left to the operator or according to the patient’s preference.

### Image analysis

All baseline coronary angiograms, IVUS, and OCT images were taken as routine with the application of intra-coronary nitroglycerin. The severity of stenosis and reference diameter were measured by QCA (Pie Medical Imaging, Maastricht, Netherlands), IVUS (Volcano Corporation, Alajuela, Costa Rica) or OCT (Abbott Medical, Westford, Massachusetts, United States) to determine the interventional strategy and device size. All of the procedural imaging results were carefully reviewed by two independent interventionalists not involved with the procedure.

### Outcome measures

The post-PCI follow-up clinical and laboratory data were reviewed using the institutional electronic medical record system. Angiographic, procedural, and clinical outcomes were coded according to the American College of Cardiology/American Heart Association (ACC/AHA) recommendations^10^. Major adverse cardiac and cerebrovascular events (MACCE) were defined as the composite of ischemia-driven target lesion revascularization (TLR), all-cause death, non-fatal acute coronary syndrome (ACS), stroke, or heart failure requiring hospitalization within 1 year after PCI. Ischemia-driven TLR was defined as any repeated PCI of the target lesion or bypass surgery of the target vessel, performed for restenosis or other target lesion-related complications for chest pain or a positive test for ischemia (exercise stress test, 24-h Holter monitoring, resting electrocardiographic evidence of ST-segment depression or elevation in > 1 lead, or radionuclide study showing a reversible defect).

### Statistical analysis

All data were first tested for normality using the Kolmogorov–Smirnov test. Data with normal distribution were expressed as mean ± standard deviation, and categorical data were expressed as number and percentage. Differences between proportions were calculated using the chi-square test or Fisher’s exact test. Comparisons of data between two groups were performed using the independent T test. Kaplan–Meier survival curves were plotted, and the log-rank test was used for clinical outcome analysis. Patients who were lost to follow-up and those who did not complete 1-year of follow-up were censored in the model.

Because there were substantial differences in the lesion characteristics between the DCB and DES groups, we conducted propensity score matching to balance the distribution of these lesion characteristics between the two groups. The propensity score was estimated using a multiple logistic regression model including the following possible confounding variables including lesion length, diameter, lesion severity (type B2/C) and CTO lesion. Each DCB subject was matched to a maximum of two DES patients. After propensity score matching, there were 35 DCB patients matched 35 DES patients and 12 DCB patients matched 24 DES patients. The balance of covariates between the matched groups was subsequently examined.

Kaplan–Meier survival curves were plotted, and the log‐rank test was used for comparisons of the 1-year outcomes. The Fisher’s exact test was used for comparisons of the 30-day outcomes. Univariable associations between clinical characteristics and MACCEs were assessed using univariable Cox regression. In addition, stepwise backward Cox regression analysis, using a P value ≥ 0.10 as the removal criterion, was performed for multivariable analysis. A two-sided P value < 0.05 was defined as statistically significant. Statistical analysis was performed using SPSS version 25 for Windows (SPSS Inc., IL, USA) with the R-3.3 plugin extension for the propensity score analysis (the ‘Matchit’ package).

## Results

### Patient characteristics

Between January 2018 and March 2021, a total of 5212 PCI procedures were performed at National Taiwan University Hospital. Among these, a total of 216 de novo very small vessel lesions in 205 patients were treated with either a DCB or DES (device diameter ≤ 2.25 mm) and were included in the analysis. The patients were stratified into DCB and DES groups according to the final coronary devices used. Fifty-five patients (58 lesions) received a DCB, and 150 patients (158 lesions) received a DES. The baseline characteristics were similar between the DCB and DES groups. Blood biochemistry, ultrasonographic left ventricular ejection fraction, and the number and location of diseased vessel were also similar. There was no significant difference in post-PCI medications, except for a lower percentage of uninterrupted dual antiplatelet therapy (DAPT) > 3 months in the DCB group. After propensity score matching, there were 47 patients received a DCB matched to 59 patients received a DES. The baseline characteristics remained to be well balanced between DCB and DES group except for the lower percentage of uninterrupted dual antiplatelet therapy (DAPT) > 3 months in the DCB group (Table 1).

**Table 1:** Patients characteristics.

	Before PSM			After PSM		
	DCB (n = 55)	DES (n = 150)	P value	DCB (n = 47)	DES (n = 59)	P value
**Clinical data**						
Age, years	65.2 ± 11.1	66.0 ± 10.4	0.629	65.1 ± 10.6	65.1 ± 10.0	0.990
Male gender, n (%)	44 (80.0)	123 (82.0)	0.744	39 (83.0)	48 (81.4)	0.829
Body mass index, kg/m^2^	26.5 ± 3.7	25.6 ± 3.8	0.124	26.8 ± 3.9	25.4 ± 3.9	0.071
Smoking, n (%)	23 (41.8)	54 (36.0)	0.446	21 (44.7)	21 (35.6)	0.342
Heart failure, n (%)	14 (25.5)	31 (20.7)	0.463	11 (23.4)	12 (20.3)	0.704
DM, n (%)	27 (49.1)	77 (51.3)	0.776	23 (48.9)	32 (54.2)	0.587
HTN, n (%)	50 (90.9)	125 (83.3)	0.174	44 (93.6)	49 (83.0)	0.099
Dyslipidemia, n (%)	47 (85.5)	110 (73.3)	0.069	40 (85.0)	42 (71.2)	0.089
Atrial fibrillation, n (%)	7 (12.7)	8 (5.3)	0.072	6 (12.8)	4 (6.8)	0.333
ESRD, n (%)	6 (10.9)	13 (8.7)	0.624	6 (12.8)	6 (10.2)	0.75
History of MI, n (%)	7 (12.7)	11 (7.3)	0.227	5 (10.6)	7 (11.9)	0.843
History of CVA, n (%)	3 (5.5)	12 (8.0)	0.764	3 (6.4)	2 (3.4)	0.653
History of Vascular disease, n (%)	6 (10.9)	15 (10.0)	0.849	6 (12.8)	10 (16.9)	0.550
History of CABG, n (%)	3 (5.5)	5 (3.3)	0.445	3 (6.4)	3 (5.1)	0.774
ACS presentation, n (%)	9 (16.4)	34 (22.7)	0.326	6 (12.8)	11 (18.6)	0.413
**Laboratory study**						
Hemoglobin, g/dL	13.7 ± 1.9	13.1 ± 2.2	0.122	13.7 ± 1.9	13.1 ± 2.2	0.124
Platelet, K/uL	217.5 ± 58.5	228.9 ± 79.7	0.334	216.3 ± 60.2	219.6 ± 57.5	0.773
Creatinine, mg/dL	1.5 ± 1.7	1.6 ± 2.0	0.666	1.6 ± 1.9	1.7 ± 2.3	0.689
Fasting glucose, g/dL	113.2 ± 29.4	117.3 ± 34.4	0.436	111.7 ± 25.7	123.0 ± 32.2	0.052
HbA1c, %	6.3 ± 1.2	6.5 ± 1.2	0.399	6.2 ± 1.2	6.6 ± 1.3	0.158
T-CHO, mg/dL	154.4 ± 34.2	157.6 ± 39.9	0.601	154.6 ± 33.4	162.3 ± 37.1	0.268
Triglyceride, mg/dL	131.8 ± 74.8	131.1 ± 70.9	0.951	133.6 ± 74.6	139.9 ± 74.6	0.667
LDL-C, mg/dL	88.5 ± 25.6	91.8 ± 32.7	0.504	88.7 ± 23.3	94.9 ± 30.0	0.251
HDL-C, mg/dL	45.1 ± 11.0	42.9 ± 10.6	0.200	44.9 ± 11.4	43.5 ± 9.1	0.492
LVEF, %	58.5 ± 13.4	58.6 ± 12.6	0.945	58.0 ± 13.5	59.0 ± 11.9	0.711
**Coronary angiogram**						
Radial approach, n (%)	36 (65.5)	101 (67.3)	0.800	31 (66.0)	44 (74.6)	0.332
Left main disease, n (%)	5 (9.1)	23 (15.3)	0.249	4 (8.5)	4 (6.8)	1.000
Triple-vessel-disease, n (%)	37 (67.3)	104 (69.3)	0.769	33 (70.2)	40 (67.8)	0.653
Two-vessel-disease, n (%)	14 (25.5)	32 (21.3)		10 (21.3)	16 (27.1)	
One-vessel-disease, n (%)	4 (7.3)	14 (9.3)		4 (8.5)	3 (5.1)	
**Medication after PCI**						
Beta-blocker, n (%)	40 (72.7)	103 (68.7)	0.575	35 (74.5)	36 (61.0)	0.143
Statin, n (%)	46 (83.6)	115 (76.7)	0.282	38 (80.9)	44 (74.6)	0.443
Ezetimibe, n (%)	6 (10.9)	9 (6.0)	0.232	6 (12.8)	4 (6.8)	0.333
ACEI/ARB, n (%)	33 (60.0)	81 (54.0)	0.444	28 (59.6)	30 (50.8)	0.370
Spironolactone, n (%)	4 (7.3)	13 (8.7)	1.000	3 (6.4)	7 (11.9)	0.507
ARNI, n (%)	2 (3.6)	4 (2.7)	0.660	2 (4.3)	0 (0)	0.194
Nitrates, n (%)	15 (27.3)	32 (21.3)	0.370	15 (31.9)	16 (27.1)	0.590
DAPT use (≥ 1 month), n (%)	48 (87.3)	141 (94.0)	0.112	41 (87.2)	56 (94.9)	0.159
DAPT use (≥ 3 months), n (%)	44 (80.0)	137 (91.3)	0.025	37 (78.7)	55 (93.2)	0.029
Aspirin, n (%)	53 (96.4)	139 (92.7)	0.336	45 (95.7)	55 (93.2)	0.576
Clopidogrel, n (%)	49 (89.1)	128 (85.3)	0.488	43 (91.5)	53 (89.8)	0.772
Ticagrelor, n (%)	4 (7.3)	20 (13.3)	0.328	2 (4.3)	5 (8.5)	0.459
OAC, n (%)	2 (3.6)	8 (5.3)	1.000	2 (4.3)	3 (5.1)	1.000

*DM* diabetes mellitus, *HTN* hypertension, *ESRD* end-stage renal disease, *MI* myocardial infarction, *CVA* cerebrovascular accident, *CABG* coronary artery bypass graft, *ACS* acute coronary syndrome, *T-CHO* total cholesterol, *LDL-C* low-density lipoprotein cholesterol, *HDL-C* high-density lipoprotein cholesterol, *LVEF* left ventricular ejection fraction, *ACEI* angiotensin-converting enzyme inhibitors, *ARB* angiotensin receptor blockers, *ARNI* angiotensin receptor-neprilysin inhibitor, *DAPT* dual antiplatelet, *OAC* oral anticoagulants, *PSM* propensity score matching.

### Lesion characteristics

Most target lesions were in the left anterior descending artery in both groups. Significantly more ACC/AHA type B2/C lesions (58.2% vs. 36.2%, P = 0.004) and chronic total occlusion lesions (25.9% vs. 12.1%, P = 0.024) were found in the DES group compared to the DCB group. The stenotic lesion diameter was comparable between the two groups (86.6 ± 10.0% in the DCB group vs. 87.9 ± 10.8% in the DES group, P = 0.396). The reference lesion diameter was significantly smaller in the DCB group (2.0 ± 0.1 mm vs. 2.2 ± 0.1 mm in the DES group, P < 0.001). The lesion lengths were comparable between the two groups (20.2 ± 5.1 mm in the DCB group vs. 21.7 ± 5.7 mm in the DES group, P = 0.060). More 2.0 mm devices were used in the DCB group (81.0% vs. 25.3% in the DES group, P < 0.001). Although, the device length was comparable, the proportion of device length > 30 mm was significantly higher in the DES group (25.9% vs. 10.3% in the DCB group, P = 0.014). The use of rotational atherectomy was comparable in both groups, while adjunctive scoring/cutting balloons were used more frequently in the DCB group (22.4% vs. 1.3% in the DES group, P < 0.001). IVUS or OCT was used in 27.6% of the DCB group and 34.2% of the DES group (P = 0.359). There were 66.5% lesions received balloon post-dilatation after DES deployment and mean post-dilatation balloon diameter was 2.26 ± 0.17 mm. The DCB group had significantly higher lesion residual stenosis after PCI (13.5 ± 14.7% vs 0 ± 0% in DES group, P < 0.001).

After propensity score matching, there were 50 lesions in DCB matched to 61 lesions in DES group (3 patients in the DCB group received 2 DCB PCI in separate lesions and 2 patients in the DES group received 2 DES PCI in separate lesions). The lesion characteristics remained well balanced between DCB and DES groups except for the higher percentage of scoring or cutting balloon in the DCB group and the significantly higher residual stenosis in DCB group (Table 2).

**Table 2:** Lesions characteristic and procedures of very small vessel.

	Before PSM			After PSM		
	DCB (n = 58)	DES (n = 158)	P value	DCB (n = 50)	DES (n = 61)	P value
**Very small vessel lesion location**						
LAD, n (%)	25 (43.1)	76 (48.1)	0.776	21 (42.0)	27 (44.3)	0.448
LCX, n (%)	20 (34.5)	52 (32.9)		18 (36.0)	26 (42.6)	
RCA, n (%)	13 (22.4)	30 (19.0)		11 (22.0)	8 (13.1)	
**Lesion characteristics**						
Mean Lesion length, mm	20.2 ± 5.1	21.7 ± 5.7	0.060	19.8 ± 5.2	20.0 ± 4.8	0.833
Mean lesion diameter, mm	2.0 ± 0.1	2.2 ± 0.1	< 0.001	2.1 ± 0.1	2.1 ± 0.1	0.104
Diameter stenosis, %	86.6 ± 10.0	87.9 ± 10.8	0.396	86.5 ± 10.0	86.3 ± 10.2	0.899
**AHA/ACC lesion type**						
Type A lesion, n (%)	11 (19.0)	28 (17.7)	0.016	11 (22.0)	15 (24.6)	0.207
Type B1 lesion, n (%)	26 (44.8)	38 (24.1)		22 (44.0)	16 (27.1)	
Type B2 lesion, n (%)	10 (17.2)	39 (24.7)		7 (14.0)	14 (23.7)	
Type C lesion, n (%)	11 (19.0)	53 (33.5)		10 (20.0)	6 (10.2)	
Type B2/C lesion, n (%)	21 (36.2)	92 (58.2)	0.004	17 (34.0)	30 (49.2)	0.107
CTO lesion, n (%)	7 (12.1)	41 (25.9)	0.024	7 (14.0)	12 (19.7)	0.430
**Device profiles**						
Device diameter 2.0 mm, n (%)	47 (81.0)	40 (25.3)	< 0.001	39 (78.0)	39 (63.9)	0.107
Device diameter 2.25 mm, n (%)	11 (19.0)	118 (74.7)		11 (22.0)	22 (36.1)	
Mean device length, mm	26.2 ± 7.5	26.2 ± 7.5	0.974	26.0 ± 7.4	25.0 ± 6.6	0.446
Device length > 30 mm, n (%)	6 (10.3)	41 (25.9)	0.014	4 (8.0)	7 (11.5)	0.751
**Procedures profiles**						
Rotational atherectomy, n (%)	1 (1.7)	7 (4.4)	0.685	1 (2.0)	2 (3.3)	1.000
Scoring/Cutting balloon, n (%)	13 (22.4)	2 (1.3)	< 0.001	11 (22.0)	1 (1.6)	0.001
IVUS/OCT, n (%)	16 (27.6)	54 (34.2)	0.359	14 (28.0)	18 (29.5)	0.861
Post dilatation, n (%)	0 (0)	105 (66.5)	< 0.001	0	43 (70.5)	< 0.001
Dilatation balloon diameter, mm	–	2.26 ± 0.17	NA	–	2.17 ± 0.18	NA
Residual stenosis, %	13.5 ± 14.7	0 ± 0	< 0.001	13.9 ± 14.9	0 ± 0	< 0.001
Complications, n (%)	1 (1.7)	4 (2.5)	1.000	1 (2.0)	2 (3.3)	1.000

*LAD* left anterior descending artery, *LCX* left circumflex artery, *RCA* right coronary artery, *CTO* chronic total occlusion, *IVUS* intravascular ultrasound, *OCT* optical coherence tomography, *PSM* propensity score matching.

### Procedural outcomes

The technical success rate was 100%, and no device failure was observed. One wire-induced distal coronary perforation, one vascular access complication, one periprocedural myocardial infarction, and one in-hospital subacute stent thrombosis occurred in the DES group, compared to only one periprocedural myocardial infarction in the DCB group. There were no cases of periprocedural stroke, emergent coronary artery bypass grafting (CABG), or mortality in either group. The overall procedure-related complication rates were thus comparable (2.5% in the DES group and 1.7% in the DCB group, P = 1.000). The results were consistent after propensity score matching (Table 2).

### 30-day and 1-year clinical outcomes

The incidences of MACCEs at 30 days were 3.6% in the DCB group and 2.0% in the DES group (P = 0.612). There was no ischemia-driven TLR or stroke in both groups. The incidence of all-cause death or heart failure-related admission was also comparable between the two groups. The results were consistent after propensity score matching (Table 3).

**Table 3:** 30 days outcomes after very small vessel intervention for each clinical event and MACCE.

	Before PSM			After PSM		
	DCB (n = 55)	DES (n = 150)	P value	DCB (n = 47)	DES (n = 59)	P value
Ischemia-driven TLR, n (%)	0 (0)	0 (0)	NA	0 (0)	0 (0)	NA
All cause death, n (%)	1 (1.8)	2 (1.3)	1.000	1 (2.1)	0 (0)	0.443
Non-fatal ACS, n (%)	1 (1.8)	1 (0.7)	0.466	1 (2.1)	1 (1.7)	1.000
Stroke, n (%)	0 (0)	0 (0)	NA	0	0 (0)	NA
HF related admission, n (%)	0 (0)	1 (0.7)	1.000	0	0 (0)	NA
MACCE^¶^, n (%)	2 (3.6)	3 (2.0)	0.612	2 (4.3)	1 (1.7)	0.583

^¶^MACCE: Composite endpoint including ischemia-driven TLR, non-fatal ACS, Stroke, HF related admission and all-cause death.

*TLR* target lesion revascularization, *ACS* acute coronary syndrome, *HF* heart failure, *MACCE* major adverse cardiac and cerebrovascular events, *PSM* propensity score matching.

**Figure 1:**
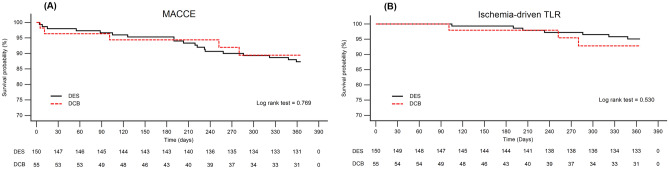
Kaplan–Meier survival estimates for clinical outcome of DCB and DES in de novo very small vessel intervention. Kaplan–Meier survival estimates for cumulative incidence of MACCE (**A**) (P = 0.769) and ischemia-driven TLR (**B**) (P = 0.530).

The cumulative incidence of ischemia-driven TLR at 1 year was 7.2% in the DCB group and 4.9% in the DES group (P = 0.530). There was no vessel thrombosis in the DCB group, and no additional stent thrombosis in the DES group. The cumulative incidence of MACCEs was 10.6% in the DCB group and 12.7% in the DES group (P = 0.769). The cumulative incidence of all-cause death, non-fatal ACS, stroke or heart failure-related admission was also comparable between the two groups. The results were consistent after propensity score matching (Table 4 and Fig. 1).

**Table 4:** One-year outcomes after very small vessel intervention for each clinical event and MACCE.

	Before PSM			After PSM		
	DCB (n = 55)	DES (n = 150)	P value	DCB (n = 47)	DES (n = 59)	P value
Ischemia-driven TLR, n (%)	3 (7.2)	7 (4.9)	0.530	3 (8.4)	2 (3.5)	0.294
All cause death, n (%)	1 (1.8)	9 (6.0)	0.287	1 (2.1)	3 (5.1)	0.538
Non-fatal ACS, n (%)	1 (1.8)	3 (2.1)	0.952	1 (2.1)	2 (3.4)	0.745
Stroke, n (%)	0 (0)	2 (1.4)	0.471	0 (0)	2 (3.5)	0.294
HF related admission, n (%)	0 (0)	3 (2.1)	0.335	0 (0)	1 (1.8)	0.433
MACCE^¶^, n (%)	5 (10.6)	19 (12.7)	0.769	5 (12.3)	7 (11.9)	0.875

^¶^MACCE: Composite endpoint including ischemia-driven TLR, non-fatal ACS, Stroke, HF related admission and all-cause death.

*TLR* target lesion revascularization, *ACS* acute coronary syndrome, *HF* heart failure, *MACCE* major adverse cardiac and cerebrovascular events, *PSM* propensity score matching.

Univariable and stepwise backward multivariable Cox regression analyses for the predictors of MACCEs at 1 year were performed. Female gender (HR 3.18 [95% CI: 1.32–7.63]; P = 0.010, ACS at presentation (HR 2.71 [95% CI: 1.20–6.11]; P = 0.016), and DAPT duration < 3 months (HR 5.90 [95% CI: 2.45–14.24]; P < 0.001) were identified as being significant predictors for MACCEs at 1 year after very small vessel PCI (Table 5).

**Table 5:** Univariable and multivariable Cox regression for predictors of MACCE.

	Univariable Cox regression		Multivariable Cox regression	
	HR (95% CI)	P value	HR (95% CI)	P value
DCB PCI	0.86 (3.22–2.31)	0.769		
Female gender	2.50 (1.07–5.85)	0.034	3.18 (1.32–7.63)	0.010
Age, years	1.00 (0.96–1.04)	0.979		
Body mass index	1.06 (0.95–1.17)	0.296		
ACS presentation	2.80 (1.24–6.30)	0.013	2.71 (1.20–6.11)	0.016
Diabetes Mellitus	2.38 (0.99–5.73)	0.054		
Heart failure	1.89 (0.81–4.42)	0.141		
Hypertension	1.95 (0.46–8.28)	0.367		
ESRD	1.44 (0.43–4.83)	0.554		
Atrial fibrillation	2.60 (0.89–7.61)	0.082		
Lesion length, mm	0.62 (0.31–1.25)	0.181		
Device diameter = 2.0 mm	0.79 (0.34–1.85)	0.591		
Device length > 30 mm	0.45 (0.13–1.50)	0.192		
ACC/AHA B2C lesion	1.19 (0.53–2.68)	0.675		
CTO lesion	0.65 (0.22–1.90)	0.432		
Statin use	0.54 (0.23–1.27)	0.157		
Beta blocker use	0.58 (0.26–1.32)	0.195		
DAPT < 3 months	4.99 (2.13–11.69)	< 0.001	5.90 (2.45–14.24)	< 0.001

*DCB* drug coating balloon, *ACS* acute coronary syndrome, *ESRD* end-stage renal disease, *CTO* chronic total occlusion, *DAPT* dual antiplatelets.

### Subgroup analysis of ACS at presentation or not

The subgroup analysis of ACS or non-ACS at presentation of the 30-day and 1-year clinical outcomes is shown in Table 6. The short-term and long-term clinical outcomes were comparable in patients with ACS or non-ACS at presentation.

**Table 6:** Short-term and longarm outcomes after very small vessel intervention for each clinical event and MACCE in ACS and non-ACS patients.

	30-day outcomes			1-year outcomes		
	DCB (n = 9)	DES (n = 34)	P value	DCB (n = 9)	DES (n = 34)	P value
**ACS subgroup**						
Ischemia-driven TLR, n (%)	0 (0)	0 (0)	NA	0 (0)	4 (13.1)	0.345
All cause death, n (%)	1 (11.1)	2 (5.9)	0.515	1 (11.1)	4 (11.9)	0.993
Non-fatal ACS, n (%)	0 (0)	0 (0)	NA	0 (0)	1 (3.3)	0.629
Stroke, n (%)	0 (0)	0 (0)	NA	0 (0)	1 (3.4)	0.678
HF related admission, n (%)	0 (0)	1 (2.9)	1.000	0 (0)	2 (6.3)	0.490
MACCE^¶^, n (%)	1 (11.1)	2 (2.9)	0.515	1 (11.1)	9 (26.5)	0.467

	30-day outcomes			1-year outcomes		
	DCB (n = 46)	DES (n = 116)	P value	DCB (n = 46)	DES (n = 116)	P value
**Non-ACS subgroup**						
TLR, n (%)	0 (0)	0 (0)	NA	3 (8.6)	3 (2.7)	0.119
All cause death, n (%)	0 (0)	0 (0)	NA	0 (0)	5 (4.3)	0.202
Non-fatal ACS, n (%)	1 (2.2)	1 (0.9)	0.489	1 (2.2)	2 (1.7)	0.796
Stroke, n (%)	0 (0)	0 (0)	NA	0 (0)	1 (0.9)	0.582
HF related admission, n (%)	0 (0)	0 (0)	NA	0 (0)	1 (0.9)	0.586
MACCE^¶^, n (%)	1 (2.2)	1(0.9)	0.489	4 (10.6)	10 (8.6)	0.718

^¶^MACCE: Composite endpoint including ischemia-driven TLR, non-fatal ACS, Stroke, HF related admission and all-cause death.

*TLR* target lesion revascularization, *ACS* acute coronary syndrome, *HF* heart failure, *MACCE* major adverse cardiac and cerebrovascular events.

## Discussion

### Main findings and previous studies

In the present real-world retrospective cohort, we clearly demonstrated comparable procedural and 1-year outcomes for very small vessel PCI between the DCB and DES groups. PCI for very small vessels (reference diameter 2.0–2.25 mm) is not uncommon in daily practice and remains a challenge^5,6^. These lesions are usually seen in patients with diabetes mellitus, female gender, older age, multi-vessel disease, peripheral vascular disease, and history of myocardial infarction or CABG^11–13^. Moreover, they are often long and diffuse, categorized as unfavorable AHA/ACC type C lesions^2^. Revascularization by CABG or PCI for very small vessel disease has been shown to be of limited clinical benefit due to anastomotic difficulty, poor distal run-off, and incomplete revascularization^14,15^. Diffuse small vessel disease is known to be related to incomplete revascularization in CABG^15^, and restenosis, incomplete revascularization, and worse outcomes in PCI^1–4^.

The higher rate of restenosis in small vessel PCI is partly explained by the inevitable neointimal hyperplasia and exaggerated relative loss in an already small vessel lumen^16^. DESs provide an antiproliferative effect, but the thick struts in older generation designs can lead to permanent luminal loss despite proper apposition. The evolution of stent technology may improve the results of small vessel PCI. A recent trial showed that a reduction in stent strut thickness resulted in lower TLR rates at 3 years^6^. A more fundamental approach may be achieved using DCBs with the concept of “leaving nothing behind”^17^, by the homogenous delivery of antiproliferative drugs to the vessel wall and avoiding permanent metallic implantation. Several prior studies have shown promising outcomes of DCBs with comparable results to those of DESs in vessels ranging from 2.75 to 3.0 mm^18–20^.

The results of DCBs should, in theory, be more favorable in smaller vessels. Previous retrospective studies have demonstrated comparable 1-year results between DCBs and DESs in vessels ranging from 2 to 2.5 mm^5,21,22^. Moreover, no vessel thrombosis occurred with DCBs. The Taiwanese National Health Insurance program only reimburses for 1 month of DAPT after DCB but 6 months after DES, and therefore the proportion of patients with > 3 months DAPT was significantly lower in the DCB group in our study. Even with this limitation, there was no vessel thrombosis in the DCB group, but one (0.6%) case of subacute stent thrombosis in the DES group. Future large-scale randomized controlled trials are needed to investigate the effect of a shorted DAPT duration.

Intravascular imaging is important in very small vessel PCI, where precise visualization of plaque morphology and vessel diameter, confirmation of adequate stent apposition and/or expansion, and checking for residual thrombus or dissection all contribute to the final outcomes. Compared with intravascular imaging, the QCA or visual estimation frequently underestimates the lesion diameter^23^. However, anatomical restrictions including a small vessel caliber relative to the device profile, distal location of the target, and difficulty in imaging device delivery are major drawbacks. In our cohort, 27.6% of the DCB group received intravascular imaging compared to 34.2% in the DES group. With advances in imaging device technology, the results of very small vessel PCI may be further improved.

The use of fractional flow reserve (FFR) may also be beneficial in PCI with a DCB-only strategy. After optimal lesion preparation, an acceptable angiography result consisting of no flow-limiting dissection, ≤ 30% residual stenosis, fully dilated lesion, thrombolysis in myocardial infarction (TIMI) flow grade 3, and an FFR > 0.8 will determine good outcomes^9^. In current study, the residual stenosis in the DCB group was low, about 13.5%, which might contribute the good clinical outcomes. In the present retrospective analysis, we could not provide information on the use of FFR. The applicability of FFR and its value in very small vessel PCI, especially when a DCB-only strategy is considered, warrants future research.

In our study, female gender, ACS at presentation and DAPT < 3 months were independent unfavorable predictors for MACCEs at 1 year. The use of a DCB or DES was not related to the 1-year outcome of very small vessel PCI. In addition, lesion characteristics, device size, and lesion complexity were also not related to clinical outcomes. Previous studies have demonstrated that, compared with men, women are at a higher risk of adverse clinical outcomes following PCI^24^. However, no previous study has clearly identified the risk of female gender in predicting very small vessel interventions. In the current study, we showed that female gender was an independent predictor of cardiovascular outcomes after very small vessel PCI. In current study, ACS at presentation was the significant predictor of worse MACCEs at 1 year. It has been well established that the ACS lesions were different to non-ACS lesions, and might lead to worse clinical outcomes^25,26^. Interestingly, the subgroup analysis of the ACS and non-ACS patients in this study still demonstrated the similar clinical outcomes in both groups but the case and event numbers were small. Further study is needed to support the DCB use in very small vessel PCI in ACS patients.

### Limitations

There are several limitations to this study. First, this was a small retrospective cohort study conducted at one tertiary center. Second, multiple DES and DCB designs were included, which may have confounded the analysis. Third, the lesion characteristics and device profiles were unbalanced between the two groups. The available sizes and recommended deployment techniques of DCBs and DESs may significantly have affected the operators’ choice in different lesion subsets. Further larger multicenter prospective randomized studies are needed.

## Conclusion

In the present study, we found similar clinical outcomes between DCBs and DESs in de novo very small vessel PCI. Female gender, ACS at presentation and short DAPT < 3 months were predictors of worse outcomes. With careful lesion selection and preparation, DCBs might be the alternative in very small vessel intervention.

## Data Availability

The datasets used and/or analyzed during the current study available from the corresponding author on reasonable request.

## Abbreviations

ACS: Acute coronary syndrome
ACC/AHA: American College of Cardiology/American Heart Association
CABG: Coronary artery bypass graft
DAPT: Dual antiplatelet therapy
DCB: Drug-coated balloon
DES: Drug-eluting stent
FFR: Fractional flow reserve
IVUS: Intravascular ultrasound
MACCE: Major adverse cardiac and cerebrovascular event
OCT: Optical coherence tomography
PCI: Percutaneous coronary intervention
PSM: Propensity score matching
RVD: Reference vessel diameter
TLR: Target lesion revascularization
TIMI: Thrombolysis In Myocardial Infarction
